# The outcome of human mosaic aneuploid blastocysts after intrauterine transfer: A retrospective study

**DOI:** 10.1097/MD.0000000000018768

**Published:** 2020-02-28

**Authors:** Baoli Hong, Yan Hao

**Affiliations:** a Department of Obstetrics and Gynecology, the First Affiliated Hospital of USTC, Division of Life Sciences and Medicine, University of Science and Technology of China, Anhui Provincial Cancer Hospital, Hefei, Anhui; b Reproductive Medicine Center, Department of Obstetrics and Gynecology, the First Affiliated Hospital of Anhui Medical University, Anhui Province Key Laboratory of Reproductive Health and Genetics, Biopreservation and Artificial Organs, Anhui Provincial Engineering Research Center, Anhui Medical University, Hefei, P.R. China.

**Keywords:** aneuploidy, biopsy, mosaicism, preimplantation genetic testing (PGT)

## Abstract

To explore whether mosaic/aneuploid embryos can be transferred when there is no normal embryo available for transplant.

The clinical pregnancy outcomes and amniocentesis outcomes of transplanted mosaic embryos during 28 preimplantation genetic testing (PGT) cycles were retrospectively analyzed. Chromosomes of 4 donated mosaic blastocysts were comprehensively screened by next-generation sequencing.

About 10 (35.7%) of the 28 transferred mosaic embryos were implanted and had a gestational sac. But 5 women miscarried due to lack of fetal heartbeat between the 7th and 12th week of pregnancy. Five women had full-term pregnancies and gave birth to 5 healthy babies. Three of the 4 donated mosaic blastocysts had normal trophectoderm and inner cell mass, but the other 1 had abnormal embryonic cell mass.

When no normal transplantable embryo is available in the PGT cycles, but the underlying risk must be fully informed.

## 1 Introduction

Human-assisted reproductive techniques in reproductive medicine have revolved successfully since the birth of the first in-vitro fertilization (IVF) baby in 1978. Then preimplantation genetic testing (PGT) emerged in 1990 when the sexes of the cleavage-stage embryos in 2 couples, both with X-linked diseases, were determined.^[1]^ PGT, including PGT for aneuploidy (PGT-A), PGT for monogenic/single gene defects and PGT for chromosomal structural rearrangements (PGT-SR), is aimed to obtain cell biopsy from a developing human oocyte or embryo in an IVF cycle.^[2]^

PGD is mainly aimed to prevent the birth of affected children from parents with known genetic abnormalities. PGS may provide a viable approach through enhanced selection to reduce the risk of adverse reproductive outcomes associated with the transfer of chromosomally abnormal embryos.^[3]^ The advent of PGT and other modern technologies enables mosaicism identification^[4]^ and draws a different ploidy status of the studied embryos.

Chromosomal mosaicism, which is commonly seen in IVF-derived human embryos, is a major influence factor on implantation failure and spontaneous miscarriages and affects the IVF success rate. At least 40% to 60% of human embryos are abnormal, and mostly (about 80%) are in the advanced maternal age, according to a systematic review involving studies that explored mosaicism through array comparative genomic hybridization (aCGH) or real-time quantitative polymerase chain reaction (qPCR).^[5]^

Mosaic embryos, characterized by the presence of a mixture of diploid and aneuploid cell lines and lying in between euploid and fully abnormal embryos, are usually not used for transfer because they are considered as abnormal.^[6]^ Nevertheless, healthy live births after mosaic aneuploid blastocyst transfer were reported in 2015 for the first time.^[7]^ However, can mosaic/aneuploid embryos be transferred when there is no normal embryo for transplant?

This study was aimed to report the outcomes of human mosaic aneuploid blastocysts after intrauterine transfer at the Reproductive Medicine Center, Department of Obstetrics and Gynecology, the First Affiliated Hospital of Anhui Medical University, and to gain knowledge about mosaic embryos.

## 2 Patients and methods

This retrospective study was conducted from July 2014 to May 2015 at the Reproductive Medicine Center, Department of Obstetrics and Gynecology, the First Affiliated Hospital of Anhui Medical University.

### 2.1 Intracytoplasmic sperm injection

In brief, after a series of routine controlled ovarian hyperstimulation processes, metaphase II oocytes were fertilized by intracytoplasmic injection of a single paternal sperm, which prevented the embryo from being contaminated by spermatozoa bounded to the zona pellucida. After incubation in 6% CO_2_ incubators for 48 hours, the numbers of cleavage cells were counted, and the cell morphology and fragments were observed on the 3rd day to assess the quality of the fertilized embryos. Then the embryos were cultivated to the 5th or 6th and 7th day to slow down the growth to the blastocyst stage. Each blastocyst was scored in terms of blastocyst formation percentage and morphological structure according to the Gardner and Schoolcraft criteria.^[8]^

Embryo biopsy was performed in the blastocyst stage in order to more accurately assess the embryonic chromosomes. First, a hole was created in the zona pellucida at the cleavage stage by using a non-contact laser, and then 5 to 10 trophectoderm (TE) cells were dissected.

### 2.2 PGT

Whole chromosome aneuploidy and segmental chromosome imbalances can be detected by testing the TE samples from blastocyst biopsies.^[9]^ Between January 2014 and December 2016, the embryos were studied using PGT (including aCGH and next-generation sequencing [NGS]) and further classified according to their chromosomal constitutions.

### 2.3 Embryo selection for transfer

Frozen embryos routinely diagnosed as normal (euploid and balanced) were thawed, and then just 1 embryo was placed in an embryo catheter and transferred to a women's uterus for implantation at proper time. If the patient had no other normal embryos available, the mosaic embryo was used for transfer. Finally, 26 women, who had no euploid embryo or balanced embryo with implantation failure, were transferred with mosaic embryos between January 2014 and September 2016.

The patients were informed with the PGT results and allowed to ask about the potential consequences of mosaic embryo transferring. We tailored the consultation according to the types of chromosomal abnormalities and informed the patients about the risks of implantation failure and miscarriage. A signed informed consent was obtained from each couple prior to the embryo transfer.

Serum β-human chorionic gonadotropin on the 14th day after transfer was detected to confirm the biochemical pregnancy, and transvaginal ultrasound examination was performed on the 30th day to confirm the clinical pregnancy. In the middle of the pregnancy period, the normality of the fetal chromosome karyotype was determined by amniocentesis.

### 2.4 Further experiment

Four mosaic embryos were donated to us for further research and divided into 2 parts of inner cell mass (ICM) and TE. Whole genome amplification of each part was re-analyzed by NGS.

## 3 Results

Twenty-six couples (average maternal age of 30.75 years, and in the range of 23 to 41 years old) undergoing PGT were enrolled. Among the total 28 oocyte retrieval cycles, 20 cycles were detected by PGT-SR to be with specific genetic abnormalities in 1 or both parents, while the remaining 8 cycles were found by PGT-A to be with advanced maternal age (≥40 years), recurrent spontaneous miscarriage with unexplained reason, and recurrent implantation failure.

Of the 408 oocytes collected, 373 oocytes were at the mature metaphase II stage and 286 oocytes were fertilized normally, which resulted in 134 high- morphologic-quality embryos for biopsy in the blastocyst stage. Whole genome amplification was successful in 129 of the 134 TE biopsies, with a failure rate of 3.7%. According to the testing time, 134 embryos were cytogenetically fixed and analyzed by either aCGH or the NGS-based 24-chromosome aneuploidy screening protocol on a highly-validated platform.

The aCGH and NGS results showed that only 19 patients had mosaic embryos and the other 7 patients had at least 1 euploid embryo available for transfer. However, the 8 euploid blastocysts with the best morphologic development were implanted unsuccessfully, indicating that the ability of an embryo to reach the blastocyst stage may not be an appropriate criterion for embryo transfer selection.

Twenty-six women, who had been fully contacted and informed about the risks of implantation failure and miscarriage, were selected for implantation (each had only 1 or 2 mosaic blastocysts available). After failure of the first mosaic embryo transplant, 2 of them received a second transplant.

The clinical outcomes studied here were slightly encouraging. A total number of 28 mosaic embryos were transferred to 26 women, of whom 11 women got pregnant (including 1 biochemical pregnancy), and 10 (35.7%) embryos were implanted and had a gestational sac. Details of the 11 cases of pregnancy are listed in Table 1. At the 30th day after embryo transfer, the clinical pregnancy in each case was confirmed via ultrasound examination, which showed 10 fetuses that had cardiac activity. However, 5 women miscarried between the 7th and 12th weeks of pregnancy, due to lack of fetal heartbeat. Our results present several limitations to be considered. Regrettably, we did not know the fetal karyotypes because the 5 miscarried women refused chorionic chromosome examination. In the future, we will pay more attention to the collection of these data. Five women with full-term pregnancies gave birth to 5 healthy babies.

**Table 1:**
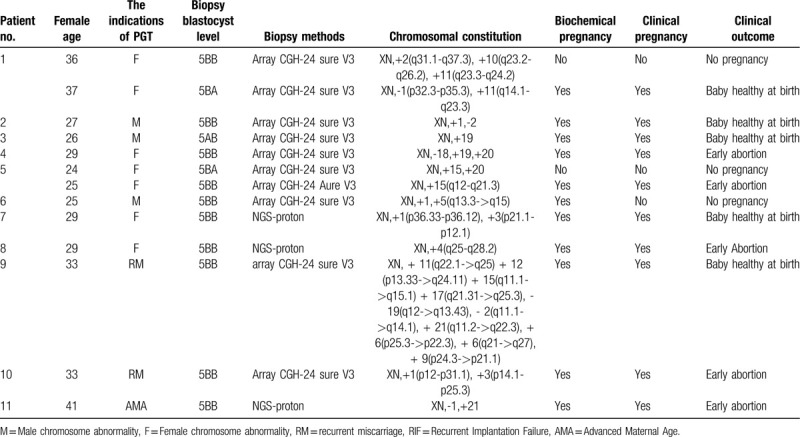
Clinical outcomes of single mosaic blastocysts transferred.

Around the 18th week of gestation, the karyotypes from cultured amniotic fluids in 4 women were checked through amniocentesis, which showed 3 fetuses with normal karyotypes and 1 with a balanced translocation karyotype (Table 2). In the remaining 1 couple with a single pregnancy, the woman refused antenatal aneuploidy screening due to the risk of miscarriage. The patient had a nearly 1-year-old child and her fetus was normal, at least phenotypically. All 5 pregnancies developed to term and gave birth to 5 singleton healthy babies.

**Table 2:**
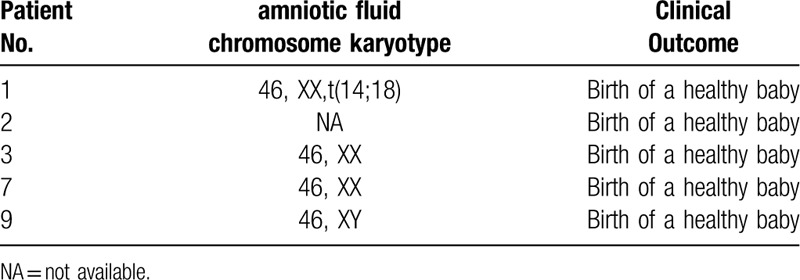
Results of amniocentesis.

The comprehensive chromosome screening of the 4 donated mosaic blastocysts was re-analyzed by NGS. Among them, 3 blastocysts had normal TE and ICM, but the other 1 had abnormal embryonic cell mass. Further details are shown in Table 3 and Figure 1  .

**Table 3:**
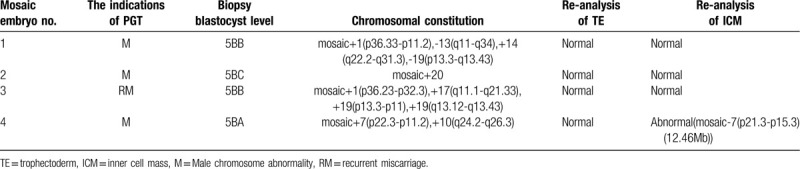
Re-analysis of 4 mosaic embryos by NGS.

**Figure 1:**
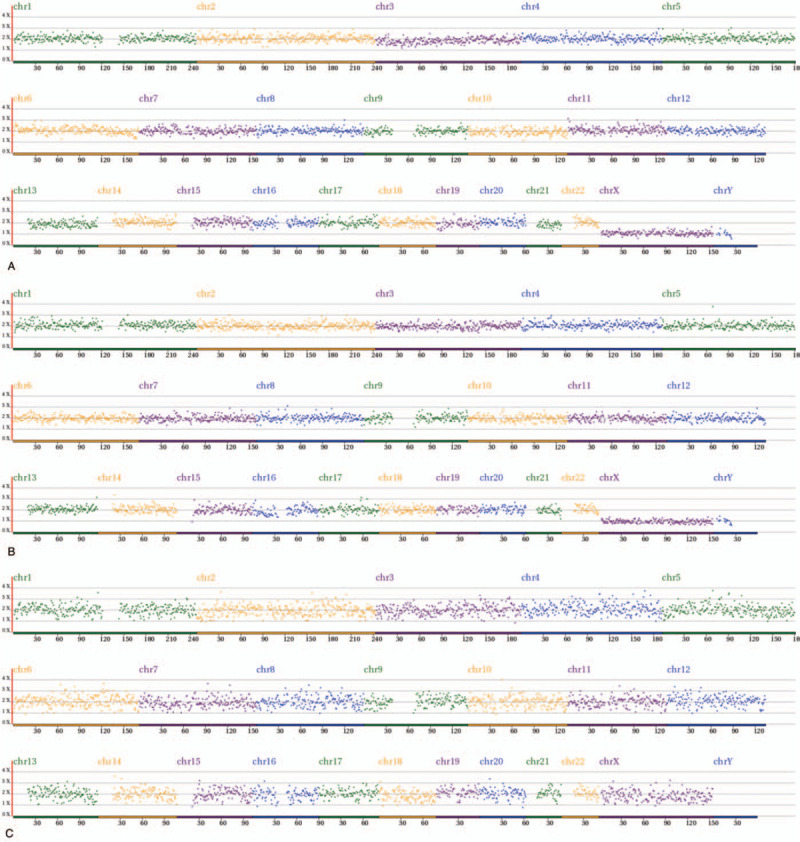
Re-analysis of comprehensive chromosome screening of 4 mosaic blastocysts by NGS. A-D: trophectoderm re-analysis results, E-H: inner cell mass re-analysis results. A-G: normal. Arrow refers to the deletion of partial fragment on chromosome 7. NGS = next-generation sequencing.

**Figure 1 (Continued):**
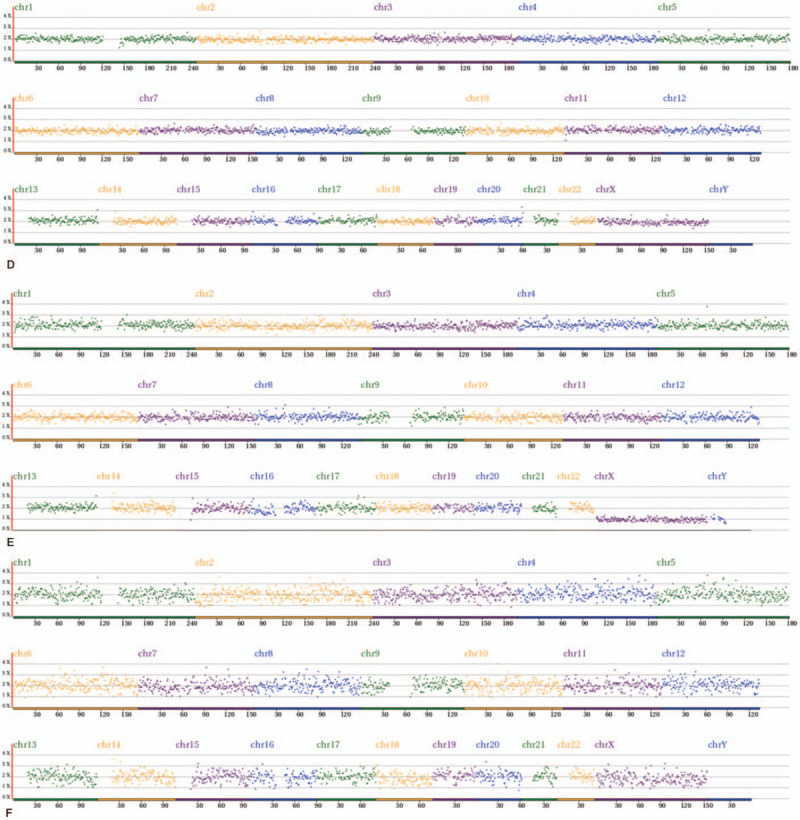
Re-analysis of comprehensive chromosome screening of 4 mosaic blastocysts by NGS. A-D: trophectoderm re-analysis results, E-H: inner cell mass re-analysis results. A-G: normal. Arrow refers to the deletion of partial fragment on chromosome 7. NGS = next-generation sequencing.

**Figure 1 (Continued):**
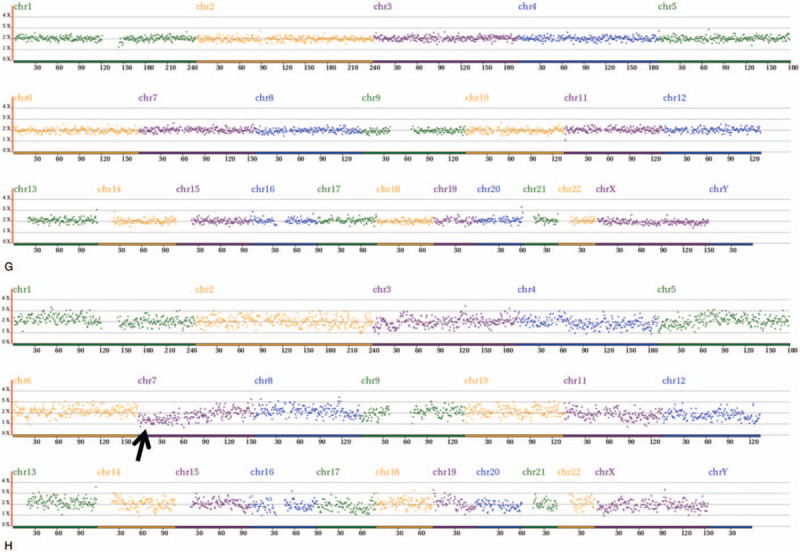
Re-analysis of comprehensive chromosome screening of 4 mosaic blastocysts by NGS. A-D: trophectoderm re-analysis results, E-H: inner cell mass re-analysis results. A-G: normal. Arrow refers to the deletion of partial fragment on chromosome 7. NGS = next-generation sequencing.

## 4 Discussion

Successful IVF is partially dependent on the successful election of viable embryos, but any abnormality in the pre-implantation stage will have more impact at later stages as the number of cells increases. Mosaicism detected during embryonic development may be confined to a particular area, and chromosomal mosaicism may significantly contribute to implantation failure and spontaneous miscarriage.^[10]^

Studies prove the blastocyst stage biopsy outperforms the cleavage stage biopsy in detecting chromosome abnormalities and mosaicism.^[11]^ Nevertheless, TE is the best stage for biopsy, as mosaicism may complicate the analysis and interpretation of the aneuploidy screening results.^[12]^ Thus all samples in the present study were obtained from blastocyst-stage embryos.

### 4.1 Primary misdiagnosis: the testing accuracy

Since no test is completely accurate, some embryos labelled as abnormal may be normal and have normal reproductive potential in practice, and vice versa. Thus, the possibility of a PGT result being incorrect is actually a question to distinguish human error or an insincere result from genetic testing. In recent years, TE biopsy and other more comprehensive and reliable analytical platforms (eg, single nucleotide polymorphism array, qPCR, and aCGH) have been validated with class I data to improve the implantation and delivery rates of many infertile people.^[13]^

Furthermore, the parallelism of NGS data provides a unique opportunity to use the DNA barcoding methodology to evaluate multiple samples for different indications on the same sequencing chip.^[14]^ Thus, NGS is the best approach for 24-chromosome aneuploidy screening. In our center, the overall clinical pregnancy rate of embryo-transferred patients detected by aCGH is 47.73%. In the past year, the clinical pregnancy rate of embryos tested by NGS was 58.06% (more specific data to be published in another article). Our results validate the accuracy of the new aCGH and NGS embryo screening methods at the blastocyst stage.

The high concordance between NGS and aCGH makes aneuploidy screening credible for routine clinical application.

### 4.2 The extent and type of mosaicism

Chromosomal mosaicism, featured by the presence of 2 or more distinct cell lines, prevails throughout human preimplantation and post-implantation development and may cause miscarriages, stillbirths or live births. Reported, there are 3 main mechanisms by which chromosomal mosaicism can lead to loss and/or gain of chromosomes, including nondisjunction, anaphase lagging, and endoreplication.^[15]^ However, the comparison of mosaicism extent with IVF outcomes suggests some mosaic embryos can develop into viable pregnancies.^[7]^ Since the number of mosaic cells is a crucial part of embryo potential, the embryos with a lower proportions of normal cells seemingly has higher developmental capacity.^[16]^ However, our center cannot provide data of the mosaicism extent, and we will pay more attention to the detection of causes in this area.

Mosaic chromosomes can be divided into different types according to several criteria, such as percentage of abnormal cells, type of chromosome abnormality (segmental, trisomy, monosomy, complex abnormal), and chromosomes involved. This division is further complicated by different rates of abnormal cells among mosaic individuals as well as by the tissues affected^[17]^ The implantation rates do not differ after the transfer of trisomic, monosomic or segmental mosaics. As reported, a notably poorer clinical outcome was observed only with the transfer of complex mosaic embryos, which had very little potential to implant^[18]^ Thus, we hypothesize that mosaicism may play a certain role in embryo development, but the role may be depending on the range and type of chromosomes involved.

The NGS-based technology with an enhanced resolution also can detect duplication or deletion in as small size as 5 mb.^[19]^ However, the vast majority of clinically meaningful duplication and deletion syndromes involve abnormalities much smaller than 5 mb, which is beyond the limit resolution of contemporary screening platforms. Furthermore, some mosaic blastocysts may be viable if we can detect the extent and type of mosaic embryos that can develop into normal healthy children. Also if the chimeric cells can be detected after TE biopsy, they may not be discarded.

### 4.3 Apoptosis

As reported, the rate of mosaicism varies broadly from 15% to 90% at the cleavage stage, but from 1% to 2% during prenatal diagnosis.^[20]^ As mentioned earlier, analytical studies on blastocyst-stage embryos show the rates of chromosomal abnormalities in the cleavage stage are higher than in the blastocyst stage.

From the cleavage stage to the blastocyst stage, some abnormal chromosomes may very likely fail to develop to blastocysts. Probably the aneuploid cells have growth defects or are destroyed by apoptosis, which reduces the number of cells during embryonic development and ultimately results in a normal fetus.

As reported, the average numbers of distinct chromosome errors per embryo in fertilized oocytes and at the cleavage stage are 2.2 and 3.3 respectively. As embryos develop to the blastocyst stage, the average number of errors significantly decreases to 1.1, which confirms the loss of abnormal cells.^[21]^ It is also indicated that not all cells of a human preimplantation embryo may be needed by the proper development into a child.

### 4.4 Self-Correction

Spare human preimplantation embryos are the sources for establishment of human embryonic stem cell (hESC) lines. Munné et al proposed to establish hESCs lines derived from chromosomally abnormal embryos^[23]^ Bolton et al cultured aneuploid embryos with fibroblasts and found the rate of normal cells increased from 12.5% on the 6th day to 47.8% on the 12th day, proving that normalization occurred before planting.^[22]^

Nowadays, MeioMapping allows the recovery of all chromosomes involved in meiotic divisions. As reported, 1 catastrophic meiosis in human oocytes, where none of the chromosome pairs were recombined and 12 pairs were miss-segregated, led to gross aneuploidy in metaphase II oocytes.^[24]^ MeioMapping also demonstrated ∼70% of errors occurred in meiosis I, but only half of the errors caused aneuploidy in oocytes, since ‘corrective’ segregation events at meiosis II may compensate for the initial errors.^[24,25]^ This result is also consistent with the chromosome analysis of polar bodies.^[26]^ and embryos.^[27,28]^ Therefore, preimplantation mosaicism has a greater consequence than post-implantation mosaicism.^[29]^

### 4.5 Can biopsied cells represent the entire embryo?

At around the 5th day after fertilization, the blastocyst is divided into 2 distinct parts of TE and ICM, which will develop to the placenta and the fetus, respectively.^[4]^ Studies suggest TE samples more accurately indicate the chromosome constitution of ICM in most cases. Thus, blastocyst-stage PGT was performed by removing the cells from the TE, without touching the ICM in order not to affect the embryo.

In our center, the chromosome karyotypes of 4 healthy babies were inconsistent with the PGT results. One possible reason is that blastomere biopsy can cause mosaicism in normal embryos. However, since the low discordance between ICM biopsy and TE biopsy even exists at the blastocyst stage, most mosaic abnormalities detected in TE samples are confined and do not represent the mosaicism of ICM. In confined placental mosaicism, placental tissues are completely trisomic, whereas the fetus is diploidy due to loss of trisomic chromosomes in the embryonic tissues.^[30–32]^ Cytogenetic prenatal diagnosis on chorionic villi involving different chromosomal mosaic cell lines is not necessarily extended to fetal tissues.^[33]^

The distribution of normal and abnormal cell lines between the fetus and the placenta depend on the timing and embryo-fetal localization in addition to the type of chromosomes. If the distribution terminates before the embryological separation of fetal and extra-fetal compartments, chromosomal mosaicism will be generalized to both the placenta and the fetus; otherwise, it will be confined to only 1 of them.^[34]^

Finally, knowing the probability of a mosaic embryo developing into a healthy live birth according to existing data is very significant. Evidence supports that mosaic embryos need more examination. Nevertheless, the transfer of diagnosed mosaic embryos may be considered in well-informed patients without PGT-identified euploid embryos. Amniocentesis examination and embryo chromosome karyotyping are needed after transplantation.

## 5 Conclusions

The blastocyst stage is the best stage for genetic testing of abnormalities, although the analysis of numerous cells cannot guarantee the omission of all mosaic embryos. Transfer of mosaic embryos, which is a major shift in current IVF practice, should be considered with extreme caution. Clinically, we can determine whether the observed mosaicism rates underlie the poor outcomes after PGT. Further studies should be focused on the origin of chromosomal mosaicism in human preimplantation embryos, the influence factors on its incidence, and the fate of mosaic embryos.

## Author contributions

**Conceptualization:** Baoli Hong, Yan Hao.

**Data curation:** Baoli Hong.

**Formal analysis:** Baoli Hong, Yan Hao.

**Investigation:** Baoli Hong, Yan Hao.

**Methodology:** Baoli Hong, Yan Hao.

**Writing – original draft:** Baoli Hong, Yan Hao.

**Writing – review and editing:** Baoli Hong.
