# The outcome of human mosaic aneuploid blastocysts after intrauterine transfer A retrospective study: Erratum

**DOI:** 10.1097/MD.0000000000031358

**Published:** 2022-10-07

In the article, “The outcome of human mosaic aneuploid blastocysts after intrauterine transfer A retrospective study”,^[1]^ which appears in Volume 99, Issue 9 of Medicine, the image of E/F/G in Figure 1 was incorrect. The correct figure is below.

**Figure 1.**
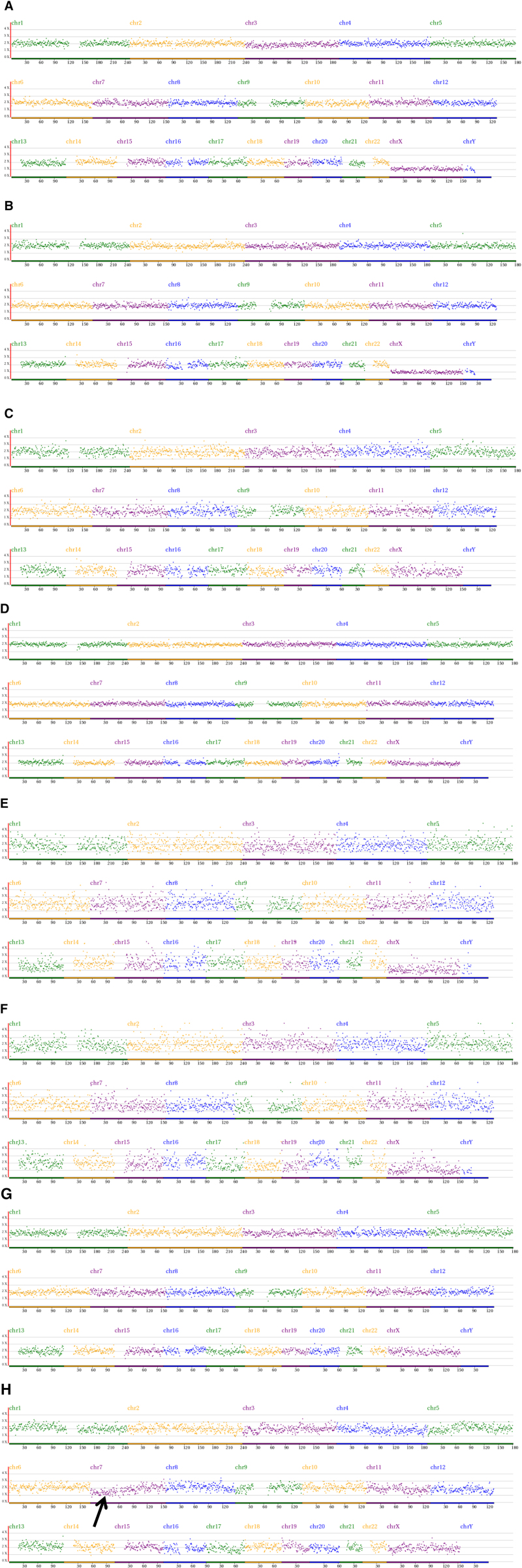
Re-analysis of comprehensive chromosome screening of 4 mosaic blastocysts by NGS. A-D: trophectoderm re-analysis results, E-H: inner cell mass re-analysis results. A-G: normal. Arrow refers to the deletion of partial fragment on chromosome 7. NGS = next-generation sequencing.
